# Psychedelic Fauna for Psychonaut Hunters: A Mini-Review

**DOI:** 10.3389/fpsyt.2018.00153

**Published:** 2018-05-22

**Authors:** Laura Orsolini, Michela Ciccarese, Duccio Papanti, Domenico De Berardis, Amira Guirguis, John M. Corkery, Fabrizio Schifano

**Affiliations:** ^1^Psychopharmacology, Drug Misuse and Novel Psychoactive Substances Research Unit, School of Life and Medical Sciences, University of Hertfordshire, Hatfield, United Kingdom; ^2^Neomesia Mental Health, Villa Jolanda Hospital, Jesi, Italy; ^3^Polyedra, Teramo, Italy; ^4^Servizio per Dipendenze Patologiche, Department of Mental Health and Pathological Dependences, AUSL, Reggio Emilia, Italy; ^5^NHS, Department of Mental Health, Psychiatric Service of Diagnosis and Treatment, Hospital G. Mazzini, Teramo, Italy; ^6^Department of Neuroscience, Imaging and Clinical Science, University G. D'Annunzio, Chieti, Italy

**Keywords:** psychedelic animals, psychedelics, NPS, novel psychoactive substances, hallucinogens, psychedelic fauna

## Abstract

Currently different classes of psychoactive substances are *easily* available for abuse, including several hundred novel psychoactive substances (NPS). Some of these drugs occur naturally in plants and animals or are chemically modified from plant or animal compounds and have been abused by humans over millennia. Recently, the occurrence of a new “drug culture” (e.g., psychonauts) who consume a great variety of NPS with hallucinogenic/psychedelic properties, facilitated the development of a new “psychedelic trend” toward the consumption of substances contained in some species of animals (“psychedelic fauna”). The present review aims at providing an overview of the most commonly abused “psychedelic animals,” by combining a dual search strategy coming from online psychonauts' experiences and English literature searches on the PubMed/Medline Google Scholar databases. A multilingual qualitative assessment on a range of websites and online resources was performed in order to identify a list of animals who possess some psychoactive properties and could be abused by humans for recreational purposes. Several species are implicated (i.e., ants, amphibians, fish). Routes of administration depend on the animal, substance included, metabolism, toxicity and individual, social and cultural variability. Online purchase and access are easy through tourism-related search strategies (“*frog trip*,” “*help of charmer snake,”* “*religious trip*”).

## Introduction

Humans have used a range of naturally occurring psychoactive substances to modify their minds, for recreational/mystic/spiritual/religious/psychedelic purposes, over millennia (1). Many psychotropic chemicals, widely distributed in plants and animals, were discovered by ancient hunter-gatherers prior to the Neolithic agricultural revolution (2). Humans have learnt how to cultivate/modify/exploit these chemicals and pass on this cultural knowledge to others (1–3). Most commonly abused natural drugs and, nowadays, novel psychoactive substances (NPS), cause changes in brain systems that alter consciousness or affect moods/emotions in some way (1–6). Moreover, ritualistic/spiritual use of these psychoactive substances has a long history among ancient tribes/shamanic communities (7–9), by suggesting some evolutionary benefits (mainly in terms of an increased chance in the survival of a species) related to the historical spread of plant- and/or animal-derived compounds possessing psychoactive properties, mostly entheogens/hallucinogens (3, 10, 11). For example, many psychotropic substances originally were taken by humans due to their bactericidal and/or antiparasitic effects (12). Within this context, some psychoactive compounds have been progressively taken by humans, despite some toxicological effects, as their intake might determine some evolutionary benefit, in terms of increasing the survival of a species and, then, increasing the reproducibility rate (1–6). Moreover, some psychoactive substances, naturally occurring in “fauna,” used for ritualistic and religious purposes over the millennia, could be taken by humans, even though they may have some toxicological effect, as the advantages in taking them would exceed the disadvantages, from an evolutionary perspective. Therefore, the spread of these psychoactive substances, naturally occurring in “fauna,” may be facilitated by some “*cultural*” processes. In fact, some drugs come with a “*cultural reputation*” for giving pleasure or relief of physical or emotional pain (13). Subsequently, in the light of the recent NPS phenomenon, the “*reputation*” of these recreational drugs in animals has also been amplified by the virtual dissemination through the Internet and social networks (3, 5, 11, 14). Moreover, the current generation of drug users (i.e., psychonauts) belong to a new sub-culture, which seems to resemble more to the shamanic communities (3, 15), by specifically possessing an “*attractiveness*” toward such entheogens/psychedelic/hallucinogenic substances, also included in some animals and/or parts of them.

Despite many psychoactive substances/NPS have been easily found in plant sources (11), a variety of animal sources of psychoactive substances appear to be equally abused, potent and risky. The term “*psychoactive fauna*” comes from the Greek noun *psuchè* (“*life breath*,” “*spirit*,” “*soul*,” “*mind*”), the Latin adjective *activus* (“*active*”), and *Fauna*, the name of the Roman goddess of fertility (16). “*Psychoactive fauna*” is currently used to denote the group of animals whose body parts or excretions contain one or more substances which, in a sufficiently high dose, have the potential to alter the user's state of consciousness (17). These compounds, naturally occurring in “fauna,” may be as well considered NPS, as the “novelty,” as previously defined, does not necessarily mean “new” psychoactive compounds, but all individual drugs in pure form or in complex preparations that are not yet scheduled under the Single Convention on Narcotic Drugs (1961) or the Convention on Psychotropic Substances (1971) (5).

As the recent spread and interest toward the “natural” NPS by the psychonauts' community, the present mini-review aims at providing an overview of the presence of some substances with psychoactive/psychedelic properties in fauna, by identifying their potential human abuse/misuse, their pharmacological and clinical effects on humans, in order to better qualify another category of NPS.

## Materials and methods

A mini-review was carried out by using the PubMed/Medline and Google Scholar databases. However, given the limitation of peer-reviewed data published so far, a preliminary nonparticipant multilingual qualitative study of a list of prodrug websites and other online resources (i.e., e-newsgroups, chat-rooms, bulletin boards, and e-newsletters) was conducted in order to obtain a list of potentially representatives of the “*psychedelic fauna*.” A systematic Internet search was conducted on Duckduckgo® and Google® which included the following keywords: “*animal's name*” and/or possible acronyms, street names etc. plus “*to buy*,” “*experience*,” “*trip*,” “*legal high*,” “*psychedelic*,” “*hallucinogen*,” “*psychoactive*.” The first 5 pages recorded per search term and search engine were consequently analyzed and selected only if relevant in terms of information and data provided regarding to the “*psychedelic fauna*.” Within the time frame January–July 2017, data were collected from 12 unique prodrug websites. Confidentiality measures applied to the dataset included storage in an online, password-protected computer and removal of screen pseudonyms, URLs, country and city identifiers. Some 2,900 fora threads were screened. After removal of those Web pages, which were either duplicates or non-relevant to the aims of the study, 268 fora threads, were analyzed and used to identify four main species implicated. Ethical approval for the study has been sought and granted by the Department of Pharmacy Ethics Committee at the University of Hertfordshire (December 15, 2010, reference code PHAEC/10-42), with a further extension of the approval granted in November 2013.

Then, we combined the search strategy of free text terms and exploded MESH headings for the topics of Psychedelic Fauna and Novel Psychoactive Substances as following: ((((*Psychedelic* OR *hallucinogenic* OR *psychoactive) substances) [*Title/Abstract]) AND ((various name of *Animals*) *[*Title/Abstract]))), as previously identified with the above-mentioned online search. Secondary searches were performed using the reference list of included articles and relevant systematic reviews. All articles published in English without time restriction were selected. Studies published through to 15 September 2017 were included. We considered studies describing some psychoactive/psychedelic/hallucinogenic effects following the intake of some animals (or parts of them), through different routes of administration, by humans. Working independently and in duplicate, two reviewers (LO and MC) read the papers and determined whether they provided data on psychedelic fauna. To be included in the present review, studies were required to meet the following criteria: (a) empirical and peer-reviewed study; (b) at least an abstract with estimates and/or full results published in English; (c) investigate psychoactive/psychedelic/hallucinogenic properties of some animals. Studies evaluating the intake of psychedelics/hallucinogens/other psychoactive substances by animals were properly excluded as not pertinent with the aims of the present paper. Moreover, studies mainly focused on intoxications rather than psychedelic experiences following the intake of some animals were also excluded from this review. As non-systematic review, reviews, letters to editors and meta-analyses were as well considered for retrieving data. LO and MC, independently extracted the data. Disagreements were resolved by discussion and consensus with a third member of the team (DP). Data were collected using an *ad-hoc* developed data extraction spreadsheet. The present comprehensive review provides summary of data collected on three main categories of animals (fish, amphibians and ants), as illustrated in Table 1.

**Table 1:** Summary of results.

**Species**	**Origin**	**Psychopharmacological effects**	**References**
**HALLUCINOGENIC FISH**			
a) Clown fish and damselfish (sp. *Abudefduf septemfasciatu*s; commonly called “*Banded sergeant*”) b) Rabbitfish (sp. *Siganus argenteus*; sp. *Siganus corallinus*; sp. *Siganus luridus*; sp. *Siganus rivulatus*; sp. *Siganus spinus*) c) Sea bream (sp. *Sarpa salpa*, commonly called ‘*Salema*’) d) Sea chub (sp. *Kyphosus inerascens*; sp. *Kyphosus vaigiensis*; *Kyphosus bigibbus*) e) Surgeon fish (sp. *Acanthurus triostegus*, commonly called ‘*Convict surgeonfish*’) f) Goatfish (sp. *Mulloidichthys flavolineatus*; sp. *Upeneus taeniopterus*) g) Mullet (sp. *Migil cephalus*; sp. *Neomyxus leuciscus*) h) Groupers (sp. *Epinephelus corallicola*, commonly called ‘*Coral grouper*’)	South Africa and Hawaiian and Norfolk Islands in the Pacific Ocean. The *Sarpa salpa* originates from temperate and tropical areas, from the Atlantic coast of Africa extending to the Mediterranean Sea, particularly near to Spanish coasts; occasionally found around the British coastline.	Fish contain hallucinogenic substances. **If ingested raw:** may induce hallucinatory and onyroid effects such as vivid/ terrifying auditory and visual hallucinations, dizziness, loss of equilibrium, lack of motor coordination and mental depression, terror and nightmares, itching, burning of the throat, muscular weakness, rarely abdominal distress. **If orally ingested:** “*Sarpa salpa*” fish may produce vivid auditory and visual hallucinations.	(18–22)
Sea chubs from the genus *Kyphosus*, supposed to be *K. Fuscus* or more likely *K. Vaigiensis*	Norfolk Island, between Australia and New Zealand	Hallucinations and ‘*dreadful nightmares*’	(18)
*Urolophus jamaicensis* species	The Caribbean and Colombia	Entheogen/intoxicant/inebriating and aphrodisiac effects originating from stingrays' venom	(23)
*Siganus spinus*	Waters around Réunion, South Atlantic	Psychedelic effects	(24)
*Mulloides flavolineatus*	Hawaii	Psychedelic effects	(24)
*Tetraodontidae* include puffers, balloon fish, blowfish, bubble fish, globefish, swellfish, toadfish, and toadies.	Tropical regions of South America, Africa and South East Asia	**If orally ingested:** poisonous puffer fish can cause a slight numbness of the lips and tongue, followed by increasing paresthesia in the face and extremities, sensations of lightness or floating. Headache, epigastric pain/nausea/diarrhea and/or vomiting may also occur. Reeling or difficulty in walking have been reported. The second stage of intoxication includes increasing paralysis, respiratory distress, altered speech, dyspnea, cyanosis, and hypotension. Whilst paralysis increases, convulsions, mental impairment, cardiac arrhythmia and death may occur.	(25, 26)
*Fugu*	Japan	Stimulant and aphrodisiac effects **If orally ingesting a non-lethal dose (i.e.**,<**8** μg per kg body weight)**:** tingling in the lips, fingers, and toes, and extremities may occur. **If orally ingesting a lethal dose (i.e.**, >**8** μg per kg body weight)**:** numbness, anesthesia, paresthesia, abdominal pain, nausea, and vomiting, muscle paralysis and respiratory insufficiency may occur.	(27, 28)
*Somniosus microcephalus*	North Atlantic and Artic Oceans	**If orally ingested:** may cause diarrhea/ vomiting/ hallucinations/ numbness. It may cause a state of near-death for several days, while the subject remains conscious.	(29)
Sea sponges such as *Smenospongia aurea*	Caribbean Sea	Psychedelic effects	(30–32)
Sea sponges such as *S. echina*	Caribbean Sea	Psychedelic effects	(30–32)
Sea sponges such as *erongula rigida*	Western Atlantic Ocean: Florida, Gulf of Mexico, and the Caribbean	Psychedelic effects	(30–32)
**PSYCHEDELIC AMPHIBIANS**			
*Bufo alvarius* and *Bufo marinus*	Mesoamerica	Used as ritual intoxicants owing to their viscous milky-white venom that contains bufotenin and bufotoxin. **If orally ingested:** *Bufo* toad venom can be fatal. Single deep inhalations of vaporized venom can produce intense and transient psychoactive effects mainly auditory and visual hallucinations.	(33)
*Bufo marinus*	North America	*B. Marinus* venom was used as “*Zombie's powder*”. **If orally ingested:** *Bufo* toad venom can be fatal. Single deep inhalations of vaporized venom can produce intense and transient psychoactive effects mainly auditory and visual hallucinations.	(34, 35)
*B. Alvarius*	The Sonoran Desert an area of California across the southern half of Arizona and South Mexico.	It contains the enzyme O-methyl-transferase, which converts bufotenin (5-OH-DMT) to the potent hallucinogen 5-MeO-DMT. The skin also contains bufotenin analogs. **If orally ingested:** *Bufo* toad venom can be fatal. Single deep inhalations of vaporized venom can produce intense and transient psychoactive effects mainly auditory and visual hallucinations.	(36, 37)
*Phillomedusa bicolor*	The Peruvian and Brazilian Amazon	**Buccal absorption** of opioid peptides scraped from the skin may induce rapid pulse/incontinence/vomiting, a state of listlessness and euphoria.	(37, 38)
**PSYCHEDELIC ANTS**			
Red harvester ants (e.g., *Pogonomyrmex californicus*)	South and South Central California	**Oral ingestion** of live ants may cause hallucinogenic and/or mind-altering effects.	(39, 40)

## Results

### “hallucinogenic” fish

Certain species of fish, particularly coming from South Africa, in the Hawaiin and Norfolk Islands in the Pacific Ocean, have been demonstrated to contain hallucinogenic substances which may give a “*fishing trip*” like that produced by lysergic acid diethylamide (LSD) intake (34). Toxic fish species belonging to eight families have been implicated: (a) Clown fish and damselfish (sp. Abudefduf septemfasciatus; commonly called “*Banded sergeant”*); (b) Rabbitfish (sp. *Siganus argenteus*; sp. *Siganus corallinus*; sp. *Siganus luridus*; sp. *Siganus rivulatus*; sp. *Siganus spinus*); (c) Sea bream (sp. *Sarpa salpa*, commonly called “*Salema*”); (d) Sea chub (sp. *Kyphosus inerascens*; sp. *Kyphosus vaigiensis*; *Kyphosus bigibbus*); (e) Surgeon fish (sp. *Acanthurus triostegus*, commonly called “*Convict surgeonfish*”); (f) Goatfish (sp. *Mulloidichthys flavolineatus*; sp. *Upeneus taeniopterus*); (g) Mullet (sp. *Migil cephalus*; sp. *Neomyxus leuciscus*); (h) Groupers (sp. *Epinephelus corallicola*, commonly called “*Coral grouper*”) (18).

Several hallucinatory and onyroid experiences, also called “*Ichthyoallyeinotoxic or hallucinatory mullet poisoning*” or “*ichthyoallyeinotoxism*,” have been reported after ingestion of the above-mentioned fish as raw (19–21). The effects of eating Ichthyoallyeinotoxic raw fish may include vivid/terrifying auditory and visual hallucinations, dizziness, loss of equilibrium, lack of motor coordination and mental depression, terror and nightmares, itching, burning of the throat, muscular weakness, rarely abdominal distress (19–21). Symptomatology may occur within a few minutes to 2 h after consumption, and may last for up to 24 h (18, 19). The first symptoms usually comprise imbalance, loss of coordination and a generalized malaise, followed by delirium, visual and/or auditory hallucinations (mainly zooptic), depression and nightmares (18, 19, 22, 41). However, there is no clear evidence of an intentional recreational use of these toxins for their “dream-inducing” properties, as most cases have been described occurring due to an accidental intoxication (18).

The “*Sarpa salpa*” (aka “*salema porgy*,” “*dream fish*” or “*nightmare fish*”) may produce vivid auditory and visual hallucinations if orally ingested (18, 20). *Sarpa salpa* is easily recognized by its gold stripes running along its side. It usually inhabits temperate and tropical areas, from the Atlantic coast of Africa to the Mediterranean Sea, particularly near to Spanish coasts; whilst it is occasionally found around the British coastline (22). It belongs to the Sparidae family and represents a popular dish across many Mediterranean countries (22). It may cause vivid hallucinations in few minutes after ingestion, which may last for days (18). It originally became a recreational drug during the Roman Empire in which it was commonly called as “*the fish that makes dreams*” in Arabic (18). Anecdotal online trip reports described that “*[…] the subjective effects are evident the next day after eating, they had vivid nightmares (I like a giant black dog chasing me through a forest as a kid sometimes after eating some of these usually just fried whole, after being scaled and gutted on each side in butter), more lethargy than any psychedelia and with excessive consumption (usually with beer) people often have slurred speech and slow/reduced reflexes […]”* (42).

Other species commonly claimed to be capable of producing hallucinations include several species of sea chub from the genus *Kyphosus*, supposed to be *K. fuseus* or more likely *K. vaigiensis*, which may cause “*dreadful nightmares*” (18). Furthermore, some Caribbean natives, particularly Mayan tribes during the pre-Colombian period, were and are usual consumers of the *Urolophus jamaicensis* species with their stingrays' venom for their entheogen/intoxicant/inebriating and aphrodisiac properties (23). Moreover, *Siganus spinus* (aka “*the fish that inebriates*”), in the waters around Réunion, and *Mulloides flavolineatus* (formerly *Mulloidichthys samoensis*), called “*the chief of ghosts*” in Hawaii, have been consumed due to their psychedelic properties (24).

Another hallucinogenic fish group is represented by the *Tetraodontidae* fish which include puffers, balloon fish, blowfish, bubble fish, globefish, swellfish, toadfish, and toadies (25). The name derived by the tetrodotoxin (TTX) that is a particularly potent neurotoxin, which specifically blocks voltage-gated sodium channels on the surface of nerve membranes (43). Its use has been historically documented as a pain-killer, for rheumatism/arthritis/neurological pain, as aphrodisiac/inebriant and essential component used during the preparation process of the “*zombie drug”* (34). TTX is commonly present in the gonads, liver, intestines, and skin of pufferfish (43). The first symptomatology consists in a slight numbness of the lips and tongue, appearing between 20 min to 3 h after eating poisonous pufferfish. Subsequently, an increasing paresthesia in the face and extremities, which may be followed by sensations of lightness or floating, appears. Headache, epigastric pain/nausea/diarrhea and/or vomiting may occur. Occasionally, some reeling or difficulty in walking have been described. The second stage of the intoxication is an increasing paralysis. Many victims are unable to move; even sitting may be difficult. There is an increasing respiratory distress, altered speech, dyspnea, cyanosis, and hypotension. Whilst paralysis increases, convulsions, mental impairment and cardiac arrhythmia may occur. Although completely paralyzed, subject may be conscious and completely lucid until shortly before dying. Death usually occurs within 4 to 6 h, with a known range of about 20 min to 8 h (26). Puffer fish toxins have been described by psychonauts as causing “*local paralysis, apparently, a feeling similar to lidocaine*”, “*a tingly feeling on the tongue after eating the meticulously prepared puffer-fish” and for a few people there is no recreation value and plenty of risk for overdose*” (30). A few online anecdotal reports described the substance “*Zombinol*”, a fictional substance, to refer to what Wade Davis wrote in his book “*The Serpent and the Rainbow*” (44) which has been supposed to be TTX, the deadly pufferfish toxin (45, 46).

An example of fish containing TTX is *Takifugu* and related genera (27). *Fugu* fish is eaten in Japan and is famous for being deadly if prepared incorrectly. The goal of preparing *Fugu* is not to remove the drug, but to reduce its levels in the animal, so it is enjoyable to the person eating it. It has powerful stimulant and aphrodisiac effects. The trick in preparing *Fugu* is to remove just enough of the organs that contain the nerve toxin TTX. When a non-lethal dose is consumed, it causes tingling in the lips, fingers, and toes, and extremities. Whilst after a time (or with a larger dose), the toxin can cause numbness and anesthesia, paresthesia along with abdominal pain, nausea, and vomiting until muscle paralysis and respiratory insufficiency (28).

Another hallucinogenic fish is *Somniosus microcephalus*. It contains in its flesh trimethylamine oxidase which is converted into trimethylamine when eaten (29). After its oral intake, subjects described the onset of a “*shark sick*” comprising diarrhea/vomiting/hallucinations/numbness. Despite it not always being lethal, it may cause a state of near-death for several days, while the subject remains conscious (29).

Moreover, as previously discussed by Shulgin and Shulgin (1997), who wrote about “marine tryptamines” (i.e., 5-Bromo-DMT and 5,6-dibromo-DMT), there are several sea sponges like *Smenospongia aura, S. echina* and *Verongula rigida* which have been demonstrated to have some psychedelic activity (30–32).

### “psychedelic” amphibians

Overall, it has been well documented that amphibian skin may contain a large range of biological active alkaloids, most possessing a unique pharmacological and therapeutic profile (31). Amongst alkaloids identified are: steroidal *salamandarines* (from salamanders); *batrachotoxins* (a potent and selective activator and ligand for Sodium Channels); *histrionicotoxins* (potent not-competitive blockers and ligands for nicotine receptor channels); *epibatidine* (with a potent anti-nociceptive activity at nicotinic receptors); the neotropical poison frogs (*dendrobatidae*); the *pumiliotoxin* (with myo-/cardio-tonic activity) contained in some anuran genera from Dendrobatidae, Mantellidae, Bufonidae and Myobatrachidae families; several izidines (pyrrolizidines, indolizidines, quinolizidines and legmizidines), pyrrolidinesm piperidines and various tricyclics (related in structures to the coccinellines), and spiropyrrolizidines, pseudophrynamine, and the tryptamine bufotenin (47–49).

Furthermore, high levels of amines, including serotonin, histamine and tyramine, have been found in the skin of various toads and frogs and synthesized by the amphibian itself due to their irritant properties on buccal tissue which is used as chemical defense (50). In addition, high levels of vasoactive peptides such as bradykinin, sauvagine, physaelaemin, caerulein, bombesin, dermorphins, etc., have been presumably used as defense against predators and microorganisms (51).

Amongst the amphibians, *Bufo alvarius* and *Bufo marinus*, morphologically similar, possess prominent parathyroid glands that secrete a viscous milky-white venom (52) containing two cardiac glucosides, namely bufotenin and bufotoxin (33). Some anthropologists described ancient peoples of Mesoamerica who used these toads as a ritual intoxicant (7, 53, 54). Furthermore, it has been assumed to be a specific psychoactive ingredient in *B. Marinus* venom, used as “*Zombie's powder*”, known during the pre-Columbian period in North-America (34, 35). Toad venom may be sometimes found in some Chinese products used in traditional Chinese medicine, such as “*chan su*,” sold as topical aphrodisiacs (aka “*stone*,” “*love stone*,” “*rock hard*”) (55). *B. alvarius*, the Sonoran Desert toad, is a semi-aquatic amphibian found only in the Sonoran Desert, an area of California across the southern half of Arizona and South Mexico (36). *B. Alvarius* contains the enzyme O-methyl-transferase, which converts bufotenin (5-OH-DMT) to the potent hallucinogen 5-methoxydimethyl-tryptamine (5-MeO-DMT) (36). The skin contains some indolealkylamines and their metabolites belonging to the common series of 5-hydroxy-indolealkylamines (e.g., bufotenin) and to the less common series of 5-methoxyindolealkylamines (e.g., O-methyl-bufotenin) (37, 56, 57). In addition, its skin contains also sulfur-containing indolealkylamines (e.g., bufoviridine). Consuming *Bufo* toad venom orally, through licking or eating, is ineffective and potentially risky, being associated sometimes with fatalities, as the venom was evolved to be a defensive poison to deter predators from eating the toads. Furthermore, many species of frogs and toads produce other venoms or skin irritants that should not be ingested orally. Single deep inhalations of vaporized venom proved powerfully psychoactive effects within 15 s. Consistent with the known effects of 5-MeO-DMT, the intoxication is intense and short-lived, marked by auditory and visual hallucinations. The strongest effects dissipated after 5 min, but residual changes in perception persisted for up to 1 h (35, 36).

Another specie is *Phillomedusa bicolor*, which is a large green nocturnal frog that lives in the trees of the Peruvian and Brazilian Amazon. Adult frogs secrete a material which is used by the native Mayoruna Indians as a hunting aid (37). Skin secretions are rich in vasoactive, opioid peptides and a peptide called “adenoregulin” (58). Peptides tested acted as potent *mu* opioid agonists on isolated organ preparations (59). Skin secretions, previously scraped from a live frog and stored dry on a stick mixed with saliva, for buccal absorption, may induce a plethora of symptoms including rapid pulse/incontinence/vomiting, followed by the onset of a state of listlessness (which may last some days) that proceeds with a euphoric state (38).

### Psychedelic ants

It has been as well documented that ants have been used in both curative and preventative medicine, for treating common illnesses (e.g., paralysis, gastrointestinal illnesses, severe colds, pain, arthritis, and gynecological disorders), frequently swallowed live as an emetic or bitten the exterior of the ants' body (60). Behind the medicine practice, ants seemed to play a significant role during some initiatory/ritual/esoteric activities. For example, ants have a prominent role during the antinic or “*ant ordeal*” of the Luiseiio, a rite which followed temporally the ritual of *Datura* drinking (39). The ingestion of live ants may cause hallucinogenic and/or mind-altering effects, as described in discussions on the shamanistic behavior or medical knowledge of native people (39). Indian people ate harvester ants after 3 days of abstaining from food, water, and sex and avoiding contact with blood, as a ritualistic hallucinogen (40). They ate balls of moistened eagle down with about 5 ants inside each (38, 60). The dose was regulated, from dozens to ninety or so balls, and the ant feeding stopped when the eyes of the subject turned red, they became lethargic and refused more (40). To obtain some shamanistic powers, the ants would be eaten in a similar manner, every summer, until the powers were obtained (40). Ant ingestion persisted through the Mission Period (1800–1878), but these practices appeared to have been abandoned during the last century (40). However, most studies were confounded by the simultaneous employment of techniques such as fasting, sleep deprivation, and the concomitant use of *Datura* and/or other psychotropic substances.

However, it has been speculated that the ants used in vision quests may have belonged to the yellow honey ant or other species of the *Myrmacomecocystus* genus, which do not contain any known psychoactive substances (39). *Red harvester ants* (e.g., *Pogonomyrmex californicus*), largely used in religious and medical practices, were as well reported to be taken by native people of Southern and South-central California, as hallucinogens (40). Their venom contains many kinds of proteins, enzymes, histamines, and other chemicals. It has been reported to be 5 and 8–10 times more toxic than, respectively, Oriental Homet and honeybee venoms (61). The doses employed in visionary contexts by California Indians were clearly within the range of pharmacological activity, representing approximately 35% of a lethal dose for an individual with a body weight of 45.5 kg (40). Moreover, it contains formic acid and polypeptide kinins which induce pain/inflammation/hypotension. Some kinins own nicotinic cholinergic activity that may be responsible for the induction of hallucinations (40).

## Discussion

The present paper specifically focused on invertebrates and vertebrates, such as ants, fish, and amphibians and their possible hallucinogenic properties and their relative and potential human recreational consumption and/or abuse. Specifically, the above-mentioned “*psychoactive fauna”* (17) have been reviewed, focusing on their first intake according to a historical perspective, their chemical/pharmacological profile, potential mechanisms of action and desired/adverse effects. Despite limited literature and scientific evidence being available to date, as most studies more specifically focused on psychedelic/hallucinogenic drugs contained in some plants and plant-derived, a plethora of data coming from the online platform and psychonauts' fora and/or blogs have been collected, according to a nonparticipant netnographic methodological approach, about the “*psychedelic animals”* (15) and subsequently compared with published literature, following a mini-review approach.

Substances influencing mood and thinking processes have been known to humanity at least from early Neolithic times in all known cultures (8). Entheogens are typically psychedelics/hallucinogens, and, by definition, imply the use of psychoactive substances for religious/spiritual reasons (9, 62, 63). Entheogens generate transcendental feelings or hallucinatory experiences, which may be enhanced by ritualistic environments e.g., darkness, chanting, spiritual discourse. Shamans throughout the world incorporate entheogens in their arsenal of techniques to connect with the spirit world (64).

Nowadays, many different classes of psychoactive substances are available for abuse, including several hundred NPS (5, 6, 65). Some of these drugs are entirely synthetic and others occur naturally in plants (or in animals), or are chemically modified from plant or animal compounds (11). Moreover, the current drug culture, i.e., psychonauts, consume a great variety of NPS with hallucinogenic/psychedelic properties, including those contained in some species of animals (3, 15). Specifically, there is a subcategory of e-psychonauts, as previously studied, who are particularly interested in taking only “natural” NPS, like plants and animals (3, 6, 11, 15).

“*Psychedelic fauna”* appears to be divided in two groups: the producers and the consumers. Producers represent animal species able to produce psychoactive chemicals, usually by converting precursors which they consume, into a new substance able to be “psychoactive” to humans. Consumers comprise any animal which takes psychoactive chemicals and store them unchanged (42). In the present mini-review we focused on the “producers,” supposed to be taken by the psychonauts' communities due to their supposed psychedelic/hallucinogenic properties. Data were collected and summarized for three main species (i.e., fish, amphibian and ants), even though the recreational and psychedelic intake of other animals by psychonauts has been as well documented (30, 34, 42).

Amongst fish, despite the toxic agents implicated remaining unknown, some authors suggested that their hallucinogenic properties may derive from alkaloids of the indole group, dimethyltryptamine (DMT) which naturally occur in certain types of microalgae (*Caulerpaceae* family) that fish usually ingest and phytoplankton and which are probably present in the head of some fish (18, 19).

The metabolic source of TTX, included in the *Tetraodontidae* fish (e.g., puffer-, balloon-, blow-, bubble-, globe-fish, etc.) is uncertain. No algal source has been identified. TTX was recently assumed to be a metabolic product of the host. However, recent reports of the production of TTX/anhydro-TTX by several bacterial species, including strains of the family *Vibrionaceae, Pseudomonas* sp., and *Photo bacterium phosphoreum*, point toward a bacterial origin of this family of toxins. These are relatively common marine bacteria that are often associated with some marine animals. An association between TTX-producing bacteria and higher organisms appears to offer clear advantages to both partners. The bacteria get a safe place to live, eat, and reproduce; the hosts use the toxin for predation or defense or both (25, 26, 43).

Whilst amongst amphibians, the tryptamine *bufotenin*, isolated in the skin of some species of toads, has been implicated to determine hallucinogenic effects after the intake of some amphibians, as abovementioned (47–49, 51). In fact, its name originates from the *Bufo* genus of toads that secrete bufotoxins from their parotid glands (33, 52). Bufotenin is chemically like the psychedelic psilocin (4-HO-DMT), 5-MeO-DMT, and DMT (36, 56, 57).

Furthermore, some potentially hallucinogenic and/or mind-altering substances have been isolated and demonstrated to be effective from ant toxins (39, 40, 61), even though formally detected specific substances have not been implicated yet.

In conclusion, the recent re-emergence of a specific “appealing”/“attractiveness” toward psychedelic/hallucinogenic substances by psychonauts (3, 5, 15), particularly plant-derived as previously investigated (11), is gradually opening to the development and spread of a new “psychedelic trend,” including “*psychedelic fauna*” as well. Further evaluation/isolation/identification of substances potentially psychedelic/hallucinogenic from some species of animals as well as further netnographic studies, specifically investigating the psychonauts' preferences and interest toward the “psychedelic fauna” should be carried out, to better understand this new NPS trend.

## Author contributions

LO, MC, and DP: conceived the topic of the manuscript, while LO, MC, and DD carried out the main analysis; DP and AG: assisted in either screening of the studies or preparation of the attachments; JC: served as study reviewer; FS: served as senior study reviewer. All the coauthors substantially contributed to the present piece of work before approving it for final submission.

### Conflict of interest statement

The authors declare that the research was conducted in the absence of any commercial or financial relationships that could be construed as a potential conflict of interest.
